# Somatic and psychiatric health burden of male and female older incarcerated adults in Switzerland: a retrospective cross-sectional study

**DOI:** 10.1136/bmjph-2025-004164

**Published:** 2026-06-25

**Authors:** Helene Seaward, Andrea Meyer, N. Zoe Hilton, Peter Wermuth, Michael Liebrenz, Monika Egli-Alge, Tobias Vogel, Elmar Habermeyer, Lutz-Peter Hiersemenzel, Dominique Marcot, Corinne Devaud Cornaz, Elger Bernice Simone, Tenzin Wangmo

**Affiliations:** 1 Institute for Biomedical Ethics, University of Basel, Basel, Switzerland; 2 Department of Psychology, Division of Clinical Psychology and Epidemiology, University of Basel, Basel, Switzerland; 3 Waypoint Research Institute, Waypoint Centre for Mental Health Care, Penetanguishene, Ontario, Canada; 4 Dalla Lana School of Public Health, University of Toronto, Toronto, Ontario, Canada; 5 Department of Psychiatry, University of Toronto, Toronto, Ontario, Canada; 6 Aargau Psychiatric Services Central Campus Brugg-Windisch - Areal Königsfelden, Windisch, Switzerland; 7 Department of Forensic Psychiatry, Medical Faculty, University of Bern, Bern, Switzerland; 8 Forio AG, Winterthur, Switzerland; 9 Forensic Department, University Psychiatric Clinic Basel, Basel, Switzerland; 10 Medical Faculty, University of Basel, Basel, Switzerland; 11 Forensische Psychiatrie und Psychotherapie, Psychiatrische Universitätsklinik Zürich, Zürich, Switzerland; 12 Solothurner Spitaler AG, Solothurn, SO, Switzerland; 13 Filière légale, Centre Neuchâtelois de Psychiatrie Préfargier, Marin-Épagnier, Switzerland; 14 Centre de Psychiatrie Forensique, Réseau Fribourgeois de Santé Mentale, Villars sur Glâne, Switzerland; 15 University of Basel, University of Basel, Basel, Switzerland; 16 Center for Legal Medicine, University of Geneva, Genève, Switzerland

**Keywords:** Public Health, Mental Health, Preventive Medicine, Preventive Psychiatry

## Abstract

**Introduction:**

Incarcerated populations are ageing, yet their specific needs remain unclear, hindering adequate care planning. We provide a comprehensive overview of health burden among incarcerated adults in Switzerland, focusing on age-related differences.

**Methods:**

This is a retrospective cross-sectional analysis based on data extracted from medical records from 10 prisons and forensic-psychiatric institutions in Switzerland (N=384), comprising 151 older adults. We performed descriptive statistics for all variables. A multiple logistic regression analysis was conducted to identify diagnostic characteristics that differentiate older from younger incarcerated adults. We assessed whether a breakpoint exists in the association between age and total somatic disease burden using a segmented regression model.

**Results:**

Almost all older adults had at least one chronic condition (97%), of which the most common were substance use (20.4%) and mood disorders (14.3%), as well as endocrine and metabolic (25%), cardiovascular (17%) and respiratory (14%) diseases. Age differences emerged for affective (OR 1.58, 95% CI 1.23 to 2.05) and personality disorders (OR 2.06, 95% CI 1.60 to 2.66). We found an estimated breakpoint at age 39, at which the number of chronic somatic diseases began to increase at a rate greater than before.

**Conclusions:**

The increase in chronic somatic diseases around age 39 may warrant further examination of the commonly used age cut-off of 50. Given the high prevalence of chronic conditions among older adults, health services should shift from a focus on acute care to long-term chronic disease management, enhance screening for early disease detection and provide preventive care.

**WHAT IS ALREADY KNOWN ON THIS TOPIC**

- The proportion of older adults in prisons and forensic psychiatric institutions is increasing. As the subgroup with the most significant healthcare needs, they face a considerable risk of inadequate provision of services.

**WHAT THIS STUDY ADDS**

- This study confirms a high burden of disease among incarcerated adults, even within a high-resource context such as Switzerland. It further identifies age 39 as an optimal cut-off point for the increase in chronic somatic conditions in this population.

**HOW THIS STUDY MIGHT AFFECT RESEARCH, PRACTICE OR POLICY**

- This study underscores the necessity of implementing preventive measures and long-term care solutions in secure settings.

## Introduction

Older adults are the most rapidly growing age group in prison populations,^1^ and despite being a minority group, their growing numbers pose new challenges for prison systems. This demographic change mirrors the overall ageing of the population but is mainly driven by harsher sentencing practices leading to long periods of incarceration, more restrictive parole policies and an increase in life and indeterminate sentences.^2^ Further, the general prison population experiences worse health status compared with community-dwelling adults,^3^ and these disparities intensify as incarcerated persons age.^2 4^ There is a lack of comprehensive understanding of the specific health needs of older incarcerated persons, making it difficult to assess current and future healthcare demands. Having such knowledge is essential in providing healthcare that is in accordance with the principle of equivalence of care and in using prisons as a public health opportunity.^5^

A major challenge for prisons is the disease burden of their older inhabitants. For instance, over 80% of older incarcerated persons present with high rates of chronic health problems such as cardiovascular and musculoskeletal problems.^6^ The most noteworthy difference between younger and older age groups in prisons is the shift from acute to chronic health issues,^7^ underlining the variation in healthcare services that the prison system must be equipped to provide. For chronic somatic health issues, a meta-analysis reported that among older incarcerated adults, the most common problems were impaired vision (59%), cardiovascular diseases (38%), arthritis (37%), hearing loss (31%) and back pain (26%).^8^

In international reviews of older incarcerated adults, the prevalence of any psychiatric disorder ranged between 13.6% and 68.4%.^1 9^ When comparing younger and older age groups in prisons, it is noticeable that affective disorders are more common among older adults, with a pooled prevalence for depression of 19.2%. In contrast, personality disorders (17.8%) and psychotic symptoms (7.2%) were less prevalent.^10 11^ Substance use disorders were the most common mental health diagnoses among older adults with highest pooled prevalence rates for alcohol abuse (36.5%) followed by other substances (26.4%).^10^ Additionally, only few older adults consumed multiple substances simultaneously while this was common for the younger cohort.^11^ Further, the burden of dual diagnosis, defined as co-occurring mental illness and substance use disorders, is particularly high among people in prisons^12 13^ but reliable data on dual diagnosis specific for older incarcerated adults is lacking.

Incarcerated female adults are a minority in prisons comprising 6.9% of the world prison population and data about them are exceptionally scarce.^8 10^ In the data available, gender differences are most consistently pronounced for substance use disorders (14, 15) and dual diagnoses (13), with female adults having disproportionately higher prevalence rates than male adults. Additionally, it is estimated that 50–70% of incarcerated female adults have experienced abuse and violence^14^ with higher prevalence of posttraumatic stress disorders (6.2% in male and 21.1% in female).^15^ While a systematic assessment of gender differences in the older age group is lacking, preliminary findings suggest that older female incarcerated persons experience worse physical and mental health than their male counterparts.^16^

Despite prevalence rates varying substantially across studies, there is consensus that older incarcerated adults have a higher disease burden in comparison with younger incarcerated adults and same-aged adults living in the community^8 10 13^; however, studies have used varying age cut-offs to define an older person in prison, ranging between 40 and 65 years.^4^ The earlier decline in health status of prison populations compared with those in the community is attributed to unfavourable lifestyle behaviours before imprisonment (drug misuse, stressful life experiences, reduced access to medical care, personal neglect, etc) and the negative effects of imprisonment itself (psychological distress due to separation from social networks, risk of victimisation, risk of isolation, sedentary behaviour, unhealthy diet, etc).^4 17 18^

Although Switzerland has a relatively low incarceration rate of approximately 73 per 100 000 inhabitants, its prison population is ageing: the proportion of incarcerated individuals aged 49 and older has risen from 6.6% in 1984 to 15.8% in 2023.^19^ It consequently provides a unique setting to examine the health characteristics of the older prison population in comparison with younger adults. Realising that prison health is public health and ageing of the prison population necessitates careful research, the national funding body supported this project to study this population for almost a decade. This project has resulted in many important findings on older incarcerated persons in prisons (https://data.snf.ch/grants/grant/166043). This article stems from the project and gives a novel and comprehensive assessment of the prevalence of non-communicable diseases, dual diagnosis, substance use and suicide attempts among older incarcerated adults in Switzerland. We report prevalence rates categorised by younger and older persons in prison, and also provide rates by gender and sentence type (definite vs indefinite sentencing forms). We further explore the relationship between age and health burden. To our knowledge, this is the first study to provide nationwide data on the chronic health issues of older incarcerated adults in Switzerland and the first study internationally to provide information on the dual diagnosis of older incarcerated adults.

## Methods

This article follows the Strengthening the Reporting of Observational Studies in Epidemiology (STROBE) reporting guidelines and presents a retrospective cross-sectional analysis of prevalence rates of mental illness and chronic somatic diseases among persons incarcerated in Swiss prisons and forensic psychiatric institutions based on medical records. Please find the STROBE checklist in the supplementary material.

### Sample

We contacted all Swiss institutions that detained persons with long-term sentences of a minimum of 1.5 years. This included institutions that detained persons sentenced to an indefinite sentence (Art. 59, 60 and 64) or definite sentence (Art.40 (Swiss Criminal Code (SCC)). For this reason, we excluded remand prisons, juvenile detention centres and administrative detention centres. Importantly, we included institutions from the two major language regions, German and French-speaking Switzerland. We excluded the Italian-speaking region (one prison). Please see the flow chart for included institutions for more details (online supplemental figure 1).

10.1136/bmjph-2025-004164.supp2 Supplementary data

Data were collected on a rolling basis between 16 November 2018 and 28 September 2020. For each participant, entries from the past 12 months in these paper-based medical records were included. The requirement for informed consent for study participation was waived, and an opt-out procedure was implemented. Before the start of data collection, prison health or social services informed their prison population about the study and their rights to opt-out of data collection. The sharing of information differed slightly between institutions due to structural and organisational reasons. In some institutions, incarcerated persons were informed using a flyer that was visibly presented to all prisoners in the cafeteria and medical services. The flyers were presented in three different languages: German, French and English. In other prisons, such central location to inform all prisoners was not available. On these occasions, members of the research team provided information sessions to inform incarcerated persons through sharing information at their workplaces within the institutions. Every person who did not wish to participate put their name down on an opt-out sheet. Participants did not receive any compensation.

The final sample size for all subjects in this study was 384. A total of 151 older incarcerated adults participated, of whom 136 were housed in prisons, which corresponds to 15.2% of all persons aged 50 and above in Swiss prisons in the year 2020.^19^ We used the age of 50 as a cut-off to distinguish between younger and older incarcerated adults, as it falls in the middle of the range of most definitions of older incarcerated adults and is the most common cut-off used in previous research.^4^

### Patient and public involvement

Patients and/or the public were not involved in the design, or conduct, or reporting, or dissemination plans of our research.

### Variables

All data were collected from medical records by five research assistants using a data extraction document specifically developed for this study. The medical records were stored in prison health departments or at the forensic psychiatric services. The research assistants collected the data on-site using paper and pencil. They were trained in using the data extraction sheet, and regular meetings were set up to clarify any questions that arose. Alongside data collection, we conducted double entry of data into Excel files, and the harmonised version was checked by a third research team member. Data were analysed using R V.4.4.2.

We obtained demographic information (ie, age, sex, nationality and education) and collected data on mental illness and chronic somatic illnesses, categorised according to International Classification of Diseases, 10th Revision (ICD-10) codes. We inquired about suicide attempts, substance use, total disease burden, dual diagnosis burden and the level and type of treatment received. Additionally, we collected data on their conviction, which we classified according to the International Classification of Crime for Statistical Purposes.

Further, we created two larger groups of individuals serving definite and indefinite sentences because this distinction is relevant for the prevalence of mental health issues. Definite sentences refer to sentences with a time limit on the sentence (eg, Art.40 SCC). Indefinite sentences refer to sentences that have no definite end date, such as Art.59, Art.60 and Art. 64 (SCC), and are reserved for people who have mental health issues that are directly connected to the crime they committed. This category is comparable to ‘non-criminally responsible’ populations in other jurisdictions. In Switzerland, these persons are placed in prisons and forensic psychiatric institutions; for this reason, we collected data in both types of institutions.

### Analyses

We computed descriptive statistics for all variables to examine the demographic composition of the sample. We calculated frequencies and percentages for the key categorical variables, including age group, gender and sentencing type. We listed single diagnostic categories when there were three cases or more. Unadjusted ORs and corresponding 95% CIs were calculated for each variable. To interpret the ORs, we followed the recommendations proposed by Chen.^20^ In the tables, we highlight those ORs in bold that demonstrate at least a medium effect size (OR >3.47). Missing values are indicated as not available (NA) in the tables. For participant characteristics, we indicate missing values per category because the number of missing values varied across categories. In the remaining tables on diagnostic information and suicidality, no entry was treated as ‘no diagnosis’, for which reason there are no missing values indicated.

A multiple logistic regression analysis was conducted with age group (dichotomous, older vs younger) as the outcome variable and mental and somatic diseases as predictors. The purpose was to identify conditions independently associated with membership in the older age group relative to the younger group. The analysis was intended as a descriptive profile comparison and was not designed to estimate the causal effect of age on disease prevalence. For mental diseases, eleven planned predictors were initially considered; however, six were removed due to zero or low variance, leaving five predictors (ICD 10 Blocks): F10–F19, F20–29, F30–39, F40–48 and F60–69. Similarly, for somatic diseases, twenty-three predictors were initially considered, but seventeen were excluded due to zero or low variance, resulting in six retained predictors (ICD-10 chapters): 4, 6, 10, 11, 18 and 21. Prior to analysis, 11 multivariate outliers were removed from the mental disease model (final sample: n=373), and 12 multivariate outliers were removed from the somatic disease model (final sample: n=372). Multicollinearity among predictors was assessed and found to be non-problematic for both models.

Additionally, we used a segmented regression model to assess whether a breakpoint exists in the association between age and total somatic disease burden. The choice of this model was based on theoretical assumption that there may be a sudden increase in disease burden, as indicated by previous literature.^4^ However, such studies are scarce and have typically examined cut-offs within predefined age brackets. Therefore, we treated age as a continuous variable to explore the optimal cut-off. The outcome variable was square-root-transformed to improve normality. Age in years was the primary predictor, while covariates included nationality, education and marital status. The model used a null-left-slope constraint, assuming no change in disease burden before the breakpoint, with a positive linear relationship between age and disease burden thereafter. Prior to analysis, 13 multivariate outliers were identified and excluded to improve model robustness. Total somatic disease burden was operationalised as the number of distinct chronic somatic diagnoses documented for each individual at the time of data collection. This reflects a simple count of all current conditions, with each contributing equally regardless of severity. Repeated entries and hierarchically related diagnoses were collapsed into a single diagnostic entity. Conditions were classified as chronic if they were inherently defined as such in ICD-10 or explicitly described as such by the documenting healthcare professional. Minor or transient conditions were excluded unless they were specifically recorded as chronic (eg, headache).

## Results

The analysis revealed a high prevalence of both mental and chronic health conditions among the incarcerated population. Please see online supplemental table 1 for participant characteristics. A detailed account of the prevalence and unadjusted ORs are presented in online supplemental table 2 for psychiatric diagnoses and chronic somatic health issues, online supplemental table 3 for substance use and online supplemental table 4 for suicide attempts. The total burden of mental and chronic somatic diseases as well as the dual diagnosis burden is listed in online supplemental table 5.

10.1136/bmjph-2025-004164.supp1 Supplementary data

Multiple logistic regression analysis showed that, for mental illness, two predictors were positively associated with age group: ‘Mood (affective) disorders’ (*B*=0.46, *SE*=0.13, *z*=3.58, p<0.001, OR 1.58, 95% CI 1.23 to 2.05) and ‘Disorders of adult personality and behaviour’ (*B*=0.72, *SE*=0.13, *z*=5.56, p<0.001, OR 2.06, 95% CI 1.60 to 2.66). That is, the prevalence was higher in older compared with younger participants. For somatic health, ‘Symptoms, signs and abnormal clinical and laboratory findings, not elsewhere classified’ was the only variable showing higher prevalence in older compared with younger adults.

The segmented regression model revealed a breakpoint at 39.0 years (SE=5.8). The slope of the linear relationship between age and disease burden following the breakpoint was positive and significantly differed from 0 (*b*=0.020, *SE*=0.006, 95% CI 0.008 to 0.032). This is shown in the plot of fitted (red) line together with observed (jittered) values, where we assume a horizontal trend up to the breakpoint (see online supplemental figure 2).

10.1136/bmjph-2025-004164.supp3 Supplementary data

## Discussion

Our analyses revealed a high disease burden among older incarcerated adults, as approximately two-thirds of them were diagnosed with psychiatric illness and more than half were living with chronic somatic health issues. Moreover, almost all older individuals in the sample (97%) had one or more chronic conditions, which is in line with Fazel,^6^ who found a rate of 85% in English and Welsh prisons. This high disease burden can be explained by socioeconomic disadvantage, previous lifestyle factors, and the negative impact of incarceration on health.^17 18^ Although we lack surrogate measures to estimate the impact of incarceration on the health of our sample, socioeconomic status (SES) remains the most significant non-medical determinant of health and can be evaluated through indicators such as educational attainment.^21^ Our sample counts disproportionally more individuals with a low SES than the average general Swiss population, for example, only 6.2% of our sample hold a university degree, while 31.1% is the population’s average.^22^ Thus, the high disease burden could partially be explained by a large proportion of our sample coming from disadvantaged backgrounds, which is typical for prison populations.^23^

For psychiatric disorders, we found that older adults have a prevalence rate for any psychiatric diagnosis of 97% and younger adults with 57%. This difference may be due to a substantial portion of older adults serving indefinite sentences, which may have inflated our prevalence estimates, as this group, by definition, has a psychiatric diagnosis. However, with increasing age, the distribution between definite and indefinite sentences shifts: individuals with indefinite sentences are more likely to remain incarcerated for longer periods, resulting in more people growing old in prison. Consequently, a substantial proportion of older adults are serving indefinite (45%) vs definite sentences (55%).^24^ This is similar to our sample (42% definite, 40% indefinite, 17% missing), suggesting a comparable distribution to the target population. The reported prevalence rates may therefore reflect the mental health burden faced by prison healthcare systems in caring for older adults.

In line with previous literature, we identified differences in diagnostic characteristics between the age groups.^10^ For instance, we noted that for mood disorders, the prevalence for the older age group was 14.3%, whereas for the younger age group it was only 6%. The prevalence of personality disorders was higher in our sample (30.4%) compared with international literature (17.8%)^10 11^ and more common among the older age group, particularly among those diagnosed with paedophilia. This might also be due to the high proportion of adults sentenced to indefinite sentences in our sample (23.6%), among whom diagnoses of severe personality disorders were common. Another notable difference was the low rate of Attention Deficit Hyperactivity Disorder (1.6%), far below the 26.2% reported in prospective studies. This suggests possible underdiagnosis, particularly for conditions considered less relevant to psychiatric assessments or court decisions. For substance use, similar tendencies were found with older adults being more likely to use alcohol and younger adults being more likely to misuse illegal substances,^11^ particularly cannabis and cocaine. The dual diagnosis burden was similar across age groups; this information has not been reported in previous research.

Concerning gender, our findings confirm a high disease burden in female adults for both mental and somatic health^16^ and high rates for neurotic and stress-related and somatoform disorders.^15^ A surprising finding was that female adults’ psychiatric diagnoses linked to substance use and dual diagnosis burden were lower compared with previous studies in other countries.^25 26^ However, our results on substance use, without psychiatric diagnoses, indicated a high prevalence of tobacco use, alcohol consumption and the use of any illegal substance. It is unclear why, despite high substance use, female adults were less likely to have a psychiatric diagnosis for substance misuse. However, it needs to be acknowledged that the female subgroup was small (n=32), which limits the precision of the estimates. Consequently, the findings should be interpreted cautiously.

Further, the prevalence for suicide attempts of the entire sample was lower (2.6%) in comparison to an international study of prison populations (8.6%).^27^ Notably, all persons in our sample who attempted suicide had a diagnosed psychiatric disorder and half of them had a documented history of previous suicide attempts, both of which are recognised as the strongest risk factors for suicide attempts in incarcerated populations. Consistent with existing literature, a high proportion of those affected were female.^27^

Surprisingly, most of our estimates on physical health disease burden are lower than those reported in recent meta-analyses on older prisoners^8 10^ as well as in comparison to Swiss population averages.^28^ For instance, our rate for hypertension of the older age group (14%) is drastically lower in comparison with Solares, Dobrosavljevic^10^ (42.3%) and Munday, Leaman^8^ (39%). Similarly, the prevalence of hypertension of all age groups (6%) is below the average of 28.4% in the year 2022 of the Swiss general population.^28^ A similar picture can be drawn for high cholesterol, where our prevalence (2.9%) is lower than the population average of 20.3%.^28^ In contrast to this is the prevalence of diabetes, which is higher among older incarcerated adults (10%) and comparable to people at least 15 years older from the Swiss general population.^28^ This finding is concerning, as diabetes and high blood pressure, as well as high cholesterol, often co-exist,^29^ raising questions about the level of preventative screening and documentation in Swiss incarcerated adults.

Our analysis of the age at which the number of chronic somatic disease diagnoses increased at a substantially different rate revealed a cut-off at the age of 39. This age is younger than previous assumptions for drawing a distinction between younger and older incarcerated individuals. Previous studies have shown changes in rates of mental and physical disorders happening in the fifties and the impact of certain ages on the use of healthcare systems after age 52.^4^ Our findings suggest that such changes may occur at an earlier age among adults incarcerated in prisons or forensic hospitals than implied by the commonly used threshold of 50 years.^4^ This exploratory, hypothesis-generating finding does not establish a new age threshold or invalidate the 50-year cut-off. Larger, preferably longitudinal studies including all age groups, or at least adults aged 40 and older, are needed to assess their reproducibility, clinical relevance and generalisability across contexts and randomised samples.

Putting these results into broader perspective, using research findings from the general population, it is not surprising to see an increase in diagnoses after the age of 40. Extending on the example of hypertension, others have shown a positive association with age, with screening recommendations increasing after the age of 40.^30^ Additionally, there is an inverse relationship between hypertension and SES.^28^ With the prison population being predominantly composed of people with low SES, a high prevalence of hypertension and an age-wise early increase can be expected and could explain the increase in diagnoses, particularly for cardiovascular disease and related health issues, among our sample. This implies screening recommendations for the general population should be carefully evaluated for applicability to incarcerated populations, who carry a higher disease burden and may more often qualify for lower age thresholds. Given the minimal investment in early detection and intervention in carceral settings and the high screening uptake among incarcerated populations when offered,^31^ greater research and implementation efforts in this area are warranted.

Further, a large proportion of these health issues can be prevented by addressing modifiable risk factors, including obesity, diabetes, hypertension, lack of physical activity and unhealthy diet.^32^ However, prisons still don’t focus on health promotion, with high levels of physical inactivity and poor nutrition.^23^ A strong emphasis on health promotion in this hard-to-reach population could directly benefit them and reduce the demand on prison health services. If the ageing trend in carceral settings continues, healthcare planners will need to allocate more resources (staff, funding and facilities) to such preventative and long-term care. However, current services are primarily geared toward acute, short-term issues, while management of chronic diseases remains insufficient.^31^

This study has some limitations. The dataset is based on extraction of information from medical records. These were paper-based files, located in different institutions, with at times information on certain individuals distributed between prisons and forensic psychiatric services. We assured that we collected and combined data from the same individuals across the different locations. However, as can be seen in our results, only 85% of persons sentenced to an indefinite sentence had a psychiatric diagnosis. This can have two reasons: People can be sentenced to an indefinite sentence without a psychiatric diagnosis but with merely dissocial traits (Art.64, 1a, SCC). However, this is expected to be an exception and can hardly account for 15% of adults missing a diagnosis. For this reason, the second explanation is more likely to be due to lack of rigour in the medical files, for which reason we expect that a detailed account of mental health issues is missing for 14 adults.

While efforts were made to ensure consistency in data collection, for example, by applying a standardised data extraction procedure across all institutions and documenting deviations between records, no other systematic differences in record-keeping were identified. The number of missing values was explicitly reported for participant characteristics but this was not feasible for diagnostic criteria. In these cases, the absence of an entry in the medical records was interpreted as the absence of an event (eg, no diagnosis or no suicide attempt), meaning that missing data could not be distinguished from true non-occurrence. This pragmatic operationalisation, common in retrospective record-based research, implicitly assumes relatively complete and consistent documentation, which is unlikely to hold in practice. As a result, the reported prevalence rates may not fully reflect clinical reality but instead represent documented incidence. They may be influenced by factors such as access to mental healthcare, determinants of healthcare utilisation (including stigma related to mental illness) and variability in recording practices among healthcare professionals.

Further, this is not a representative sample but draws on a high proportion of the older incarcerated adults in Switzerland. We covered 15.2% of the target population (individuals aged 50 and above in Swiss prisons in 2020), which is substantial considering that this vulnerable population is especially hard to reach.^33^ Future research should consider including prospective data collection methods to assess potential underdiagnosis. Stronger national record-keeping standards would improve comparability across institutions and enable much-needed large-scale, comprehensive analyses.

To conclude, this vulnerable group presents with significant health needs. Developing a national strategy for prison health appears imperative to improve early detection and intervention, enhance record-keeping standards and expand the capacity of health services to manage long-term care. Given that a large proportion of our sample comes from disadvantaged backgrounds, secure settings provide an important public health opportunity to reach such marginalised individuals with high-need profiles.

## Data Availability

Data are available upon reasonable request. The primary data sources used in this study are available upon reasonable request. Readers who wish to access data can contact the corresponding author for additional information or specific datasets.
