# Supplementary material for: Somatic and psychiatric health burden of male and female older incarcerated adults in Switzerland: a retrospective cross-sectional study
Source: BMJ Public Health. 2026 Jun 25;4(2):e004164. doi: 10.1136/bmjph-2025-004164 (PMC13358338; doi:10.1136/bmjph-2025-004164)
Supplement: Supplementary data [file bmjph-4-2-s002.pdf]

Figure 1
Flowchart of included institutions

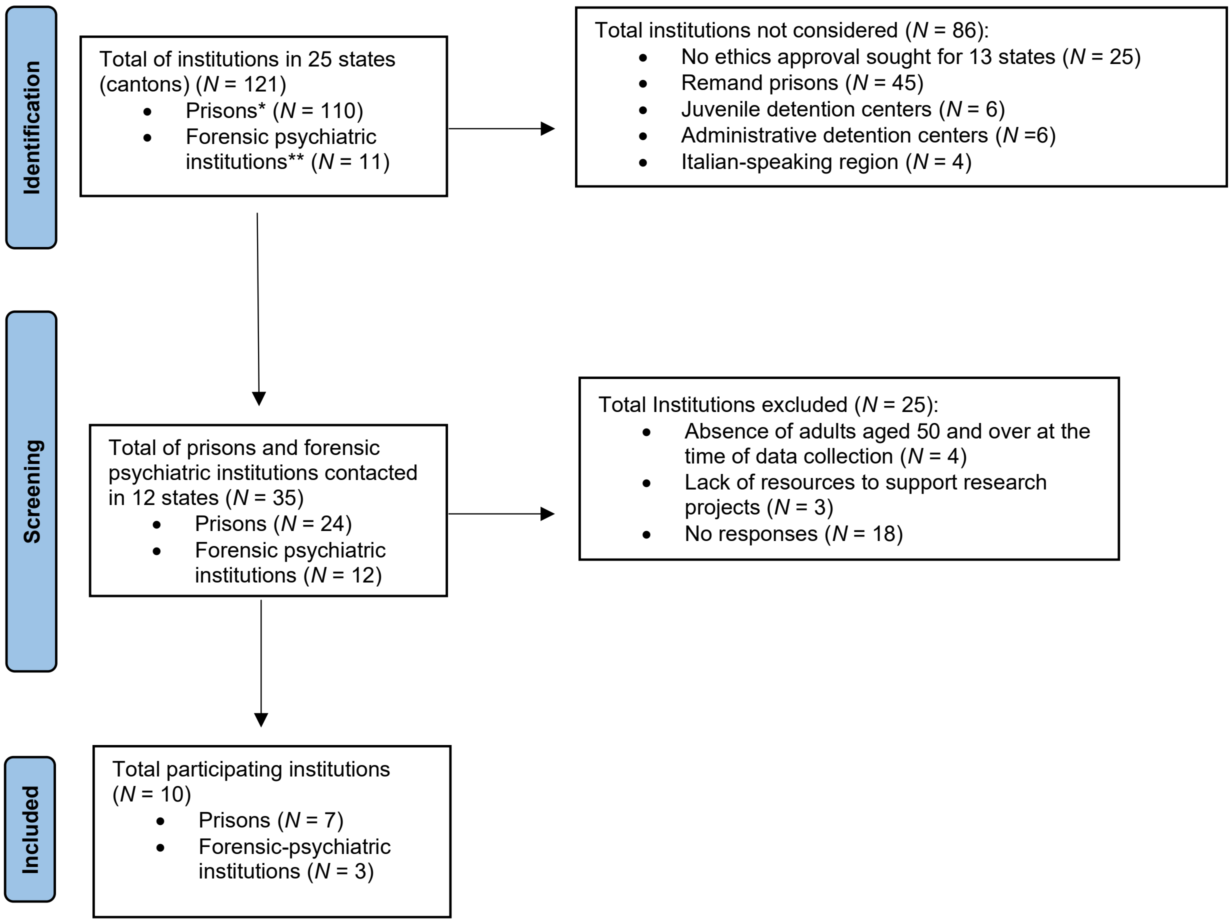

*based on the "Katalog der Justizvollzugseinrichtungen» (Swiss Directory of Prisons) from November 2016.

**based on best knowledge, there are no national figures publicly available.

Source: Page MJ, et al. BMJ 2021;372:n71. doi: 10.1136/bmj.n71.
