# Supplementary material for: Somatic and psychiatric health burden of male and female older incarcerated adults in Switzerland: a retrospective cross-sectional study
Source: BMJ Public Health. 2026 Jun 25;4(2):e004164. doi: 10.1136/bmjph-2025-004164 (PMC13358338; doi:10.1136/bmjph-2025-004164)
Supplement: Supplementary data [file bmjph-4-2-s001.pdf]

Table 1 – Participants' demographic and sentencing information

			**Sentence type (NA= 68)**		**Age (NA= 0)**		**Gender (NA= 0)**	
		**Total**	**Indefinite**	**Definite**	**50 and older**	**Under 50**	**Female**	**Male**
		***N* = 384 (100%)**	*N* = 92 (24%)	*N* = 224 (58.3%)	*N* = 151 (39.1%)	*N* = 233 (60.7%)	*N* = 32 (8.3%)	N = 352 (91.7%)
**Institution type**	High and medium-security prisons	351 (91%)	69 (75%)	214 (96%)	132 (87%)	219 (94%)	26 (81%)	325 (92%)
	Minimum security prisons	11 (2.9%)	1 (1.1%)	10 (4.5%)	3 (2.0%)	8 (3.4%)	2 (6.3%)	9 (2.6%)
	Forensic-psychiatric institution	22 (5.7%)	22 (24%)	0 (0%)	16 (11%)	6 (2.6%)	4 (13%)	18 (5.1%)
**Educational attainment**	Primary School (until 6th grade)	19 (4.9%)	4 (4.3%)	13 (5.8%)	8 (5.3%)	11 (4.7%)	2 (6.3%)	17 (4.8%)
	Secondary School (until 9th grade)	81 (21%)	21 (23%)	49 (22%)	25 (17%)	56 (24%)	6 (19%)	75 (21%)
	High School (until 12th grade)	124 (32%)	26 (28%)	76 (34%)	54 (36%)	70 (30%)	13 (41%)	111 (32%)
	Vocational training	34 (8.9%)	3 (3.3%)	24 (11%)	11 (7.3%)	23 (9.9%)	2 (6.3%)	32 (9.1%)
	University degree	24 (6.3%)	5 (5.4%)	15 (6.7%)	15 (9.9%)	9 (3.9%)	3 (9.4%)	21 (6.0%)
	NA	102 (27%)	33 (36%)	47 (21%)	38 (25%)	64 (27%)	6 (19%)	96 (27%)
**Marital Status**	Married	82 (21%)	8 (8.7%)	53 (24%)	32 (21%)	50 (21%)	7 (22%)	75 (21%)
	Single	256 (67%)	73 (79%)	162 (72%)	94 (62%)	162 (70%)	25 (78%)	231 (66%)
	NA	46 (12%)	11 (12%)	9 (4.0%)	25 (17%)	21 (9.0%)	0 (0%)	46 (13%)
**Nationality**	Swiss	123 (32%)	63 (68%)	52 (23%)	77 (51%)	46 (20%)	16 (50%)	107 (30%)
	Non-Swiss	227 (59%)	17 (18%)	166 (74%)	58 (38%)	169 (73%)	16 (50%)	211 (60%)
	NA	34 (8.9%)	12 (13%)	6 (2.7%)	16 (11%)	18 (7.7%)	0 (0%)	34 (9.7%)
**Sentencing information**								
Average length of imprisonment	Months in prison	*M=64*	*M=115*	*M=48*	*M=90*	*M=51*	*M=54*	*M=66*
	NA	104 (27%)	21 (23%)	21 (9%)	44 (29%)	41 (18%)	0 (0%)	104 (29%)
Previous incarcerations	First incarceration	214 (56%)	33 (36%)	162 (72%)	65 (43%)	149 (64%)	15 (47%)	199 (57%)
	Previously incarcerated	85 (22%)	31 (34%)	41 (18%)	42 (28%)	43 (18%)	9 (28%)	76 (22%)
	NA	85 (22%)	28 (30%)	21 (9.4%)	44 (29%)	41 (18%)	8 (25%)	77 (22%)
**Types of Offences** (based on the international classification of crimes for statistical purposes (UNODC))	Acts leading to death or intending to cause death	130 (34%)	36 (39%)	79 (35%)	60 (40%)	70 (30%)	16 (50%)	114 (32%)

Acts causing harm or intending to cause harm to the person.	96 (25%)	28 (30%)	66 (29%)	38 (25%)	58 (25%)	4 (13%)	92 (26%)
Injurious acts of sexual nature	98 (26%)	46 (50%)	45 (20%)	60 (40%)	38 (16%)	2 (6.3%)	96 (27%)
Acts against property involving violence or threat against a person.	32 (8.3%)	6 (6.5%)	19 (8.5%)	9 (6.0%)	23 (9.9%)	6 (19%)	26 (7.4%)
Acts against property only.	91 (24%)	13 (14%)	63 (28%)	20 (13%)	71 (30%)	7 (22%)	84 (24%)
Acts involving controlled psychoactive substances or other drugs.	107 (28%)	13 (14%)	76 (34%)	29 (19%)	78 (33%)	8 (25%)	99 (28%)
NA	17 (4.4 %)	3 (3.3%)	5 (2.2%)	6 (4.0%)	11 (4.7%)	0 (0%)	17 (4.8%)

Note. N = sample size (percentage). Percentages always refer to the total sample size of the respective group. *M* = mean

Table 2 – Mental health and chronic somatic health

**ICD-10 codes<br>Chapter V - Mental and Behavioural disorders**		**Sentence type (NA= 68)**				**Age (NA= 0)**			**Gender (NA= 0)**		
		**Total**	**Indefinite**	**Definite***	**OR [95% CI]**	**50 and older**	**Under 50***	**OR [95% CI]**	**Female**	**Male***	**OR [95% CI]**
**Blocks**	**Specific disorders**	***N* = 384**	*N* = 92	*N* = 224		*N* = 151	*N* = 233		*N* = 32	*N* = 352	
F00-09 Organic, including symptomatic, mental disorders		4 (1%)	3 (3.3%)	1 (0.4%)	7.52 [0.95, 153]	2 (1.3%)	2 (0.9%)	1.55 [0.18, 13.0]	0 (0%)	4 (1.1%)	0.00 [—]
F10-F19 Mental and behavioral disorders due to psychoactive substance use		**74 (19.3%)**	**25 (27%)**	46 (21%)	1.44 [0.82, 2.52]	**31 (20.4%)**	43 (18.3%)	1.14 [0.68, 1.91]	3 (9.4%)	71 (19.8%)	0.41 [0.10, 1.19]
	F10 Alcohol	33 (8.6%)	8 (8.7%)	24 (11%)	0.79 [0.32, 1.77]	17 (11.2%)	16 (6.9%)	1.72 [0.84, 3.55]	1 (3.1%)	32 (9.2%)	0.32 [0.02, 1.58]
	F12 Cannabinoids	15 (3.9%)	7 (7.6%)	8 (3.6%)	2.22 [0.76, 6.38]	5 (3.3%)	10 (4.3%)	0.76 [0.23, 2.20]	0 (0%)	15 (4.3%)	0.00 [—]
	F13 Sedatives or hypnotics	5 (1.3%)	4 (4.3%)	1 (0.4%)	**10.1 [1.47, 200]**	5 (3.3%)	0 (0%)	0.00 [—]	0 (0%)	5 (1.4%)	0.00 [—]
	F14 Cocaine	6 (1.6%)	0 (0%)	6 (2.7%)	0.00 [—]	1 (0.7%)	5 (2.1 %)	0.3 [0.02, 1.91]	0 (0%)	6 (1.7%)	0.00 [—]
	F17 Tobacco	5 (1.3%)	4 (4.3%)	1 (0.4%)	**10.1 [1.47, 200]**	2 (1.3%)	3 (1.3%)	1.03 [0.13, 6.28]	0 (0%)	5 (1.4%)	0.00 [—]
	F19 Other psychoactive substances	21 (5.5%)	8 (8.7%)	12 (5.4%)	1.68 [0.64, 4.22]	5 (3.3%)	16 (6.9%)	0.46 [0.15, 1.22]	1 (3.1%)	20 (5.7%)	0.54 [0.03, 2.71]
F20-29 Schizophrenia		34 (8.9%)	**21 (23%)**	11 (4.9%)	**5.73 [2.68, 12.9]**	16 (11%)	18 (7.7%)	1.42 [0.69, 2.88]	3 (9.4%)	31 (8.9%)	1.07 [0.25, 3.24]
	F20 Schizophrenia	24 (6.3%)	17 (18%)	7 (3.1%)	**7.03 [2.91, 18.8]**	11 (7.3%)	13 (5.6%)	1.33 [0.57, 3.06]	2 (6.3%)	22 (6.3%)	1.00 [0.16, 3.63]
	F20.0 Paranoid Schizophrenia	15 (3.9%)	11 (12%)	4 (1.8%)	**7.47 [2.48, 27.6]**	7 (4.6%)	8 (3.4%)	1.37 [0.47, 3.89]	0 (0%)	15 (4.3%)	0.00 [—]
F30-39 Mood (affective) disorders		35 (9.1%)	9 (9.8%)	20 (8.9%)	1.11 [0.46, 2.46]	**21 (14.3%)**	14 (6.0%)	2.53 [1.25, 5.24]	**6 (19%)**	29 (8%)	2.57 [0.90, 6.41]
	F31 Bipolar Disorders	7 (1.8%)	1 (1.1%)	5 (2.2%)	0.48 [0.02, 3.04]	3 (2.0%)	4 (1.7%)	1.16 [0.23, 5.34]	2 (6.3%)	5 (1.4%)	**4.63 [0.64, 22.5]**
	F32 Depressive episode	16 (4.2%)	3 (3.3%)	10 (4.5%)	0.72 [0.16, 2.42]	9 (6.0%)	7 (3.0%)	2.05 [0.75, 5.84]	3 (9.4%)	13 (3.7%)	2.70 [0.59, 8.97]
	F33 Recurrent depressive disorder	11 (2.9%)	4 (4.3%)	5 (2.2%)	1.99 [0.48, 7.69]	9 (6.0%)	2 (0.9%)	**7.32 [1.85, 48.5]**	1 (3.1%)	10 (2.9%)	1.10 [0.06, 6.05]
F40-48 Neurotic, stress-related and somatoform disorders		**40 (10.5%)**	5 (5.4%)	28 (13%)	0.40 [0.13, 0.99]	13 (8.6%)	27 (11.3%)	0.72 [0.35, 1.42]	**12 (37.3%)**	27 (7.7%)	**6.94 [3.02, 15.6]**
	F43 Reaction to severe stress, and adjustment disorders	29 (7.6 %)	2 (2.2%)	22 (9.8%)	**0.20 [0.03, 0.71]**	10 (6.7%)	19 (8.2%)	0.8 [0.35, 1.73]	10 (31%)	19 (5.5%)	**7.97 [3.23, 19.0]**
	F43.2 Adjustment Disorders	21 (5.5%)	2 (2.2%)	16 (7.1%)	0.29 [0.05, 1.04]	7 (4.6%)	14 (6.0%)	0.76 [0.28, 1.87]	7 (22%)	14 (4.0%)	**6.76 [2.38, 17.9]**
F50-59 Behavioral syndromes associated with physiological disturbances and physical factors		10 (2.6%)	3 (3.3%)	5 (2.2%)	1.48 [0.30, 6.15]	3 (2.0%)	7 (3%)	0.65 [0.14, 2.39]	2 (6.3%)	8 (2.3%)	2.87 [0.42, 12.1]
	F51 Nonorganic sleep disorders	7 (1.9%)	0 (0%)	5 (2.2%)	0.00 [—]	2 (1.3%)	5 (2.1%)	0.61 [0.09, 2.88]	0 (0%)	7 (2.0%)	0.00 [—]
F60-69 Disorders of adult personality and behaviour		**116 (30.4%)**	**59 (64%)**	53 (24%)	**5.77 [3.44, 9.85]**	**67 (44%)**	49 (21.3%)	3 [1.92, 4.72]	**10 (31.1%)**	106 (30.1%)	1.05 [0.46, 2.25]
	F60 Specific personality disorders	59 (15.3%)	27 (29%)	29 (13%)	2.79 [1.54, 5.07]	26 (17.7%)	33 (14%)	1.26 [0.72, 2.20]	6 (19%)	53 (15.3%)	1.30 [0.47, 3.12]
	F60.2 Antisocial personality disorder	27 (7.0%)	14 (15%)	12 (5.4%)	3.17 [1.40, 7.27]	8 (5.3%)	19 (8.2%)	0.63 [0.25, 1.43]	1 (3.1%)	26 (7.4%)	0.40 [0.02, 2.01]
	F60.3 Borderline personality disorder	6 (1.6%)	3 (3.3%)	3 (1.3%)	2.48 [0.45, 13.6]	2 (1.3%)	4 (1.7%)	0.77 [0.11, 3.99]	2 (6.3%)	4 (1.1%)	**5.80 [0.78, 31.0]**
	F60.8 Other specific personality disorders	15 (3.9%)	5 (5.4%)	10 (4.5%)	1.23 [0.37, 3.57]	11 (7.3%)	4 (1.7%)	**4.5 [1.51, 16.5]**	0 (0%)	15 (4.3%)	0.00 [—]
	F61 Mixed and other personality disorders	35 (9.2%)	20 (22%)	15 (6.7%)	3.87 [1.89, 8.08]	23 (15.7%)	12 (5.2%)	3.31 [1.62, 7.09]	3 (9.4%)	32 (9.2%)	1.03 [0.24, 3.13]
	F65 Disorders of sexual preference	43 (11.1%)	29 (32%)	**12 (5.4%)**	**8.13 [4.01, 17.4]**	34 (22.7%)	9 (3.9%)	**7.23 [3.49, 16.5]**	0 (0%)	43 (12.2%)	0.00 [—]
	F65.4 Paedophilia	33 (8.6%)	22 (24%)	10 (4.5%)	6.73 [3.11, 15.5]	29 (19%)	4 (1.7%)	**13.6 [5.21, 46.7]**	0 (0%)	33 (9.5%)	0.00 [—]

F70-79 Mental retardation		13 (3.4%)	**9 (9.8%)**	4 (1.8%)	**5.96 [1.89, 22.5]**	6 (4.0%)	7 (3.0%)	1.34 [0.42, 4.10]	3 (9.4%)	10 (2.9%)	3.54 [0.76, 12.3]
	F70 Mild mental retardation	10 (2.6%)	7 (7.6%)	3 (1.3%)	**6.07 [1.65, 28.6]**	4 (2.6%)	4 (1.7%)	1.03 [0.26, 3.66]	3 (9.4%)	7 (2.0%)	**5.10 [1.06, 19.4]**
F80-89 Disorders of psychological development		3 (0.8%)	3 (3.3%)	0 (0%)	0.00 [—]	1 (0.7%)	2 (0.9%)	0.77 [0.04, 8.11]	2 (6.3%)	1 (0.3%)	**23.4 [2.18, 512]**
F90-98 Behavioral and emotional disorders with onset usually occurring in childhood and adolescence		9 (2.4%)	3 (3.3%)	6 (2.7%)	1.22 [0.25, 4.75]	4 (2.6%)	5 (2.1%)	1.24 [0.30, 4.76]	1 (3.1%)	8 (2.3%)	1.39 [0.07, 7.92]
	F90 Attention-deficit hyperactivity disorders	6 (1.6%)	2 (2.2%)	4 (1.8%)	1.22 [0.17, 6.38]	3 (2.6%)	6 (2.6%)	1.55 [0.28, 8.49]	1 (3.1%)	5 (1.4%)	2.24 [0.11, 14.5]
F99 Unspecified mental disorder		0 (0%)	0 (0%)	0 (0%)	0.00 [—]	0 (0%)	0 (0%)	0.00 [—]	0 (0%)	0 (0%)	0.00 [—]
**Chronic somatic health**											
Chapter	Specific diseases										
1_Certain infectious and parasitic diseases		10 (2.6%)	1 (1.1%)	7 (3.1%)	0.34 [0.02, 1.95]	2 (1.3%)	8 (3.4%)	0.38 [0.06, 1.53]	1 (3.1%)	9 (2.6%)	1.23 [0.07, 6.86]
2_Neoplasms		4 (1.0%)	3 (3.3%)	1 (0.4%)	**7.52 [0.95, 153]**	2 (1.3%)	2 (0.9%)	1.55 [0.18, 13.0]	1 (3.1%)	3 (0.9%)	**3.75 [0.18, 30.3]**
3_Diseases of the blood and blood-forming organs and certain disorders involving the immune mechanism		5 (1.3%)	3 (3.3%)	2 (0.9%)	**3.74 [0.61, 28.8]**	2 (1.3%)	3 (1.3%)	1.03 [0.13, 6.28]	2 (6.3%)	3 (0.9%)	**7.76 [0.99, 48.6]**
4_Endocrine, nutritional and metabolic diseases		**69 (18%)**	**24 (26%)**	38 (17%)	1.73 [0.96, 3.08]	**37 (25%)**	32 (14%)	2.04 [1.21, 3.46]	**11 (34%)**	58 (16%)	2.66 [1.18, 5.71]
	E73 Lactose intolerance	5 (1.3%)	1 (1.1%)	3 (1.3%)	0.81 [0.04, 6.42]	2 (1.3%)	3 (1.3%)	1.03 [0.13, 6.28]	4 (13%)	1 (0.3%)	**50.1 [7.13, 999]**
	E00-E90 Obesity and other hyperalimentation	**37 (9.6%)**	11 (12%)	23 (10%)	1.19 [0.53, 2.50]	17 (11%)	20 (8.6%)	1.35 [0.68, 2.67]	3 (9.4%)	34 (9.7%)	0.97 [0.22, 2.91]
	E10-E14 Diabetes mellitus	17 (4.4%)	11 (12%)	4 (1.8%)	**7.47 [2.48, 27.6]**	15 (9.9%)	2 (0.9%)	**12.7 [3.52, 81.6]**	1 (3.1%)	16 (4.5%)	0.68 [0.04, 3.50]
	E78.0 Pure Hypercholesterolemia	11 (2.9%)	1 (1.1%)	8 (3.6%)	0.30 [0.02, 1.65]	7 (4.6%)	4 (1.7%)	2.78 [0.83, 10.8]	0 (0%)	11 (3.1%)	0.00 [—]
6_Diseases of the nervous system		29 (7.6%)	7 (7.6%)	16 (7.1%)	1.07 [0.40, 2.60]	17 (11%)	12 (5.2%)	2.34 [1.09, 5.16]	4 (13%)	25 (7.1%)	1.87 [0.52, 5.25]
	G40 Epilepsy	7 (1.8%)	0 (0%)	7 (3.1%)	0.00 [—]	2 (1.3%)	5 (2.1%)	0.61 [0.09, 2.88]	1 (3.1%)	6 (1.7%)	1.86 [0.10, 11.4]
	G47 Sleep disorders	8 (2.1%)	0 (0%)	6 (2.7%)	0.00 [—]	5 (3.3%)	3 (1.3%)	2.63 [0.63, 13.0]	0 (0%)	8 (2.3%)	0.00 [—]
7_Diseases of the eye and adnexa		2 (0.5%)	0 (0%)	2 (0.9%)	0.00 [—]	1 (0.7%)	1 (0.4%)	1.55 [0.06, 39.3]	1 (3.1%)	1 (0.3%)	**11.3 [0.44, 291]**
8_ Diseases of the ear and mastoid process		14 (3.6%)	6 (6.5%)	7 (3.1%)	2.16 [0.68, 6.69]	8 (5.3%)	6 (2.6%)	2.12 [0.72, 6.55]	1 (3.1%)	13 (3.7%)	0.84 [0.05, 4.44]
	H93.1 Tinnitus	6 (1.6%)	2 (2.2%)	3 (1.3%)	1.64 [0.21, 10.0]	3 (2.0%)	3 (1.3%)	1.55 [0.28, 8.49]	0 (0%)	6 (1.7%)	0.00 [—]
9_Diseases of the circulatory system		29 (7.6%)	**19 (21%)**	4 (1.8%)	**14.3 [5.18, 50.6]**	**26 (17%)**	3 (1.3%)	**15.9 [5.48, 67.8]**	1 (3.1%)	28 (8.0%)	0.37 [0.02, 1.85]
	I10-I15 Hypertensive diseases	23 (6.0%)	16 (17%)	2 (0.9%)	**23.4 [6.45, 150]**	**21 (14%)**	2 (0.9%)	**18.7 [5.36, 118]**	1 (3.1%)	22 (6.3%)	0.48 [0.03, 2.43]
10_Diseases of the respiratory system		**36 (9.4%)**	**16 (17%)**	12 (5.4%)	**3.72 [1.69, 8.39]**	**21 (14%)**	15 (6.4%)	2.35 [1.18, 4.79]	**9 (28%)**	27 (7.7%)	**4.71 [1.91, 10.9]**
	J30 Vasomotor and allergic rhinitis	9 (2.3%)	2 (2.2%)	3 (1.3%)	1.64 [0.21, 10.0]	2 (1.3%)	7 (3.0%)	0.43 [0.06, 1.82]	3 (9.4%)	6 (1.7%)	**5.97 [1.21, 23.9]**
	J44 Other chronic obstructive pulmonary disease	12 (3.1%)	7 (7.6%)	3 (1.3%)	**6.07 [1.65, 28.6]**	7 (4.6%)	5 (2.1%)	2.22 [0.69, 7.61]	4 (13%)	8 (2.3%)	**6.14 [1.56, 20.8]**
	J45 Asthma	5 (1.3%)	2 (2.2%)	2 (0.9%)	2.46 [0.29, 20.8]	4 (2.6%)	1 (0.4%)	6.31 [0.92, 124]	0 (0%)	5 (1.4%)	0.00 [—]

11_Diseases of the digestive system		35 (9.1%)	8 (8.7%)	15 (6.7%)	1.33 [0.52, 3.17]	14 (9.3%)	21 (9.0%)	1.03 [0.50, 2.08]	8 (25%)	27 (7.7%)	4.01 [1.56, 9.50]
	K59.0 Constipation	5 (1.3%)	3 (3.3%)	2 (0.9%)	**3.74 [0.61, 28.8]**	3 (2.0%)	2 (0.9%)	2.34 [0.38, 17.9]	2 (6.3%)	3 (0.9%)	**7.76 [0.99, 48.6]**
	K64 Haemorrhoids and perianal venous thrombosis	7 (1.8%)	1 (1.1%)	1 (0.4%)	2.45 [0.10, 62.4]	2 (1.3%)	5 (2.1%)	0.61 [0.09, 2.88]	0 (0%)	7 (2.0%)	0.00 [—]
	K70-K77 Diseases of liver	6 (1.6%)	2 (2.2%)	3 (1.3%)	1.64 [0.21, 10.0]	1 (0.7%)	5 (2.1%)	0.30 [0.02, 1.91]	1 (3.1%)	5 (1.4%)	2.24 [0.11, 14.5]
12_Diseases of the skin and subcutaneous tissue		16 (4.2%)	2 (2.2%)	14 (6.3%)	0.33 [0.05, 1.23]	5 (3.3%)	11 (4.7%)	0.69 [0.21, 1.94]	6 (19%)	10 (2.8%)	**7.89 [2.52, 23.0]**
	L40 Psoriasis	5 (1.3%)	1 (1.1%)	4 (1.8%)	0.60 [0.03, 4.15]	2 (1.3%)	3 (1.3%)	1.03 [0.13, 6.28]	1 (3.1%)	4 (1.1%)	2.81 [0.14, 19.7]
13_Diseases of the musculoskeletal system and connective tissue		22 (5.7%)	11 (12%)	8 (3.6%)	**3.67 [1.43, 9.78]**	16 (11%)	6 (2.6%)	**4.48 [1.80, 12.7]**	3 (9.4%)	19 (5.4%)	1.81 [0.41, 5.73]
14_Diseases of the genitourinary system		18 (4.7%)	3 (3.3%)	6 (2.7%)	1.22 [0.25, 4.75]	15 (9.9%)	3 (1.3%)	**8.46 [2.73, 37.0]**	0 (0%)	18 (5.1%)	0.00 [—]
	N40 Hyperplasia of prostate	6 (1.6%)	1 (1.1%)	2 (0.9%)	1.22 [0.06, 12.9]	4 (2.6%)	2 (0.9%)	3.14 [0.61, 22.9]	0 (0%)	6 (1.7%)	0.00 [—]
17_Congenital malformations, deformations and chromosomal abnormalities		3 (0.8%)	2 (2.2%)	1 (0.4%)	**4.96 [0.47, 107]**	1 (0.7%)	2 (0.9%)	0.77 [0.04, 8.11]	1 (3.1%)	2 (0.6%)	**5.65 [0.26, 60.5]**
18_Symptoms, signs and abnormal clinical and laboratory findings, not elsewhere classified		30 (7.8%)	10 (11%)	15 (6.7%)	1.70 [0.71, 3.90]	20 (13%)	10 (4.3%)	3.40 [1.58, 7.79]	5 (16%)	25 (7.1%)	2.42 [0.77, 6.39]
	R52.1 Chronic intractable pain	22 (5.7%)	7 (7.6%)	12 (5.4%)	1.45 [0.53, 3.74]	14 (9.3%)	8 (3.4%)	2.87 [1.20, 7.36]	3 (9.4%)	19 (5.4%)	1.81 [0.41, 5.73]
19_Injury, poisoning and certain other consequences of external causes		17 (4.4%)	7 (7.6%)	7 (3.1%)	2.55 [0.85, 7.67]	8 (5.3%)	9 (3.9%)	1.39 [0.51, 3.72]	**8 (25%)**	9 (2.6%)	**12.7 [4.41, 36.3]**
	T78.4 Allergy, unspecified	10 (2.6%)	4 (4.3%)	4 (1.8%)	2.50 [0.58, 10.8]	5 (3.3%)	5 (2.1%)	1.56 [0.43, 5.70]	4 (13%)	6 (1.7%)	**8.24 [2.01, 30.6]**
20_External causes of morbidity and mortality		2 (0.5%)	1 (1.1%)	1 (0.4%)	2.45 [0.10, 62.4]	0 (0%)	2 (0.9%)	0.00 [—]	0 (0%)	2 (0.6%)	0.00 [—]
21_Factors influencing health status and contact with health services		33 (8.6%)	3 (3.3%)	27 (12%)	0.25 [0.06, 0.72]	16 (11%)	17 (7.3%)	1.51 [0.73, 3.09]	6 (19%)	27 (7.7%)	2.78 [0.97, 6.97]
	Z65 Problems related to other psychosocial circumstances	14 (3.6%)	1 (1.1%)	11 (4.9%)	0.21 [0.01, 1.12]	8 (5.3%)	6 (2.6%)	2.12 [0.72, 6.55]	0 (0%)	14 (4.0%)	**0.00 [—]**
	Z73 Problems related to life-management difficulty	19 (4.9%)	2 (2.2%)	16 (7.1%)	0.29 [0.05, 1.04]	8 (5.3%)	11 (4.7%)	1.13 [0.43, 2.86]	6 (19%)	13 (3.7%)	**6.02 [1.98, 16.6]**
	Z73.1 Accentuation of personality traits	16 (4.2%)	2 (2.2%)	13 (5.8%)	0.36 [0.06, 1.34]	6 (4.0%)	10 (4.3%)	0.92 [0.31, 2.54]	6 (19%)	10 (2.8%)	**7.89 [2.52, 23.0]**

Note. ORs that demonstrate at least a medium effect size (OR ≥ 3.47) are highlighted, and the three highest prevalence rates for each category are shown in bold. We display the individual categories in addition to the blocks if there are 5 cases or more (based on the total column; subgroup counts may be smaller).
* Reference group for calculation of Odds Ratio
N = sample size (percentage). Percentages always refer to the total sample size of the respective group.
M = mean. SD = Standard Deviation. OR = Odds Ratio, unadjusted. CI = Confidence Interval

Table 3 - Substance use

			Sentence type (NA= 68)			Age (NA= 0)			Gender (NA= 0)		
		**Total**	**Indefinite**	**Definite***	**OR [95% CI]**	**50 and older**	**Under 50***	**OR [95% CI]**	**Female**	**Male***	**OR [95% CI]**
		***N* = 384**	*N* = 92	*N* = 224		*N* = 151	*N* = 233		*N* = 32	*N* = 352	
Tobacco use yes/no (while incarcerated)		**125 (33%)**	30 (33%)	87 (39%)	0.76 [0.45, 1.26]	37 (25%)	88 (38%)	0.53 [0.34, 0.84]	19 (59%)	106 (30%)	3.39 [1.63, 7.27]
	Average daily tobacco consumption (cigarettes smoked in a day)	*M*=16.9 (SD=12.8)	*M*=22.3 (SD=17.6)	*M*=15.4 (SD=11.6)		*M*=17.3 (SD=15.7)	*M*=16.7 (SD=11.6)		*M*=16.1 (SD=8.8)	*M*=17 (SD=13.2)	
	Age of first use	*M*=14.0 (SD=2.7)	*M*=13.6 (SD=2.6)	*M*=14.6 (SD=2.9)		*M*=15.1 (SD=2.0)	*M*=13.3 (SD=2.9)		*M*=14 (SD=4.2)	*M*=14.1 (SD=2.7)	
Alcohol yes/no (prior to incarceration)		**153 (40%)**	38 (41%)	107 (48%)	0.77 [0.47, 1.25]	55 (36%)	98 (42%)	0.79 [0.52, 1.20]	18 (56%)	135 (38%)	2.07 [1.00, 4.36]
	Alcohol involved in prior offences	17 (4.4%)	5 (5.4%)	12 (5.4%)	1.02 [0.32, 2.83]	7 (4.6%)	10 (4.3%)	1.08 [0.39, 2.89]	0 (0%)	17 (4.8%)	0.00 [—]
	Alcohol involved in index offence	24 (6.3%)	8 (8.7%)	16 (7.1%)	1.24 [0.49, 2.93]	10 (6.6%)	14 (6.0%)	1.11 [0.47, 2.55]	0 (0%)	24 (6.8%)	0.00 [—]
	Alcohol use age 18 to index incident	112 (29%)	25 (27%)	85 (38%)	0.61 [0.35, 1.03]	38 (25%)	74 (32%)	0.72 [0.45, 1.14]	20 (63%)	92 (26%)	**4.71 [2.25, 10.3]**
Any illegal drug (prior to incarceration)		**126 (33%)**	36 (40%)	82 (37%)	1.16 [0.71, 1.91]	38 (25%)	88 (38%)	0.55 [0.35, 0.87]	20 (63%)	106 (30%)	**3.87 [1.85, 8.42]**
	Age of first use	*M*=16.2 (SD=4.5)	*M*=15 (SD=2.2)	*M*=16.7 (SD=5.3)		*M*=16.5 (SD=2.1)	*M*=16.1 (SD=4.9)		*M*=12 (SD=NA)	*M*=16.5 (SD=4.6)	
	Drugs involved in prior offence	12 (3.1%)	4 (4.4%)	8 (3.6%)	1.23 [0.32, 4.00]	4 (2.6%)	8 (3.4%)	0.77 [0.20, 2.48]	1 (3.1%)	11 (3.1%)	1 [0.05, 5.40]
	Drugs involved in index offence	27 (7.0%)	6 (6.5%)	21 (9.4%)	0.67 [0.24, 1.64]	11 (7.3%)	16 (6.9%)	1.07 [0.47, 2.34]	1 (3.1%)	26 (7.4%)	0.4 [0.02, 2.01]
	Drugs use age 18 to index incident	56 (15%)	18 (20%)	38 (17%)	1.19 [0.63, 2.19]	15 (9.9%)	41 (18%)	0.52 [0.27, 0.95]	19 (59%)	37 (11%)	**12.4 [5.74, 27.8]**
Cannabis/THC		81 (21%)	32 (35%)	46 (21%)	2.06 [1.20, 3.53]	23 (15%)	58 (25%)	0.54 [0.31, 0.91]	5 (16%)	76 (22%)	0.67 [0.22, 1.67]
Cocaine		68 (18%)	14 (15%)	47 (21%)	0.68 [0.34, 1.27]	14 (9.3%)	54 (23%)	0.34 [0.17, 0.62]	4 (13%)	64 (18%)	0.64 [0.19, 1.71]
Opioids		30 (7.8%)	13 (14%)	15 (6.7%)	2.29 [1.03, 5.04]	13 (8.6%)	17 (7.3%)	1.2 [0.55, 2.53]	2 (6.3%)	28 (8.0%)	0.77 [0.12, 2.75]
Sedatives		8 (2.1%)	4 (4.3%)	4 (1.8%)	2.5 [0.58, 10.8]	6 (4.0%)	2 (0.9%)	**4.78 [1.08, 32.9]**	1 (3.1%)	7 (2.0%)	1.59 [0.08, 9.35]
Stimulants		19 (4.9%)	6 (6.5%)	11 (4.9%)	1.35 [0.45, 3.67]	7 (4.6%)	12 (5.2%)	0.9 [0.33, 2.28]	2 (6.3%)	17 (4.8%)	1.31 [0.20, 4.88]
Hallucinogens		9 (2.3%)	6 (6.5%)	3 (1.3%)	**5.14 [1.33, 24.8]**	5 (3.3%)	4 (1.7%)	1.96 [0.51, 8.03]	2 (6.3%)	7 (2.0%)	3.29 [0.48, 14.3]
Misuse of prescription drugs		12 (3.1%)	4 (4.3%)	8 (3.6%)	1.23 [0.32, 4.00]	5 (3.3%)	7 (3.0%)	1.11 [0.32, 3.53]	3 (9.4%)	9 (2.6%)	**3.94 [0.84, 14.1]**

Note. ORs that demonstrate at least a medium effect size (OR ≥ 3.47) are highlighted in bold. We display the individual categories in addition to the blocks if there are 5 cases or more (based on the total column; subgroup counts may be smaller).

* Reference group for calculation of Odds Ratio

N = sample size (percentage). Percentages always refer to the total sample size of the respective group.

M = mean. SD = Standard Deviation. OR = Odds Ratio, unadjusted. CI = Confidence Interval

Table 4 - Suicide attempts

			Sentence type (NA= 68)		Age (NA= 0)		Gender (NA= 0)	
		**Total**	**Indefinite**	**Definite**	**50 and older**	**Under 50**	**Female**	**Male**
		***N* = 384**	*N* = 92	*N*=224	*N* = 151	*N* = 233	*N* = 32	*N* = 352
Suicide attempts – before imprisonment		34 (8.8%)	12 (13.2%)	20 (8.9%)	16 (10.6%)	18 (7.8%)	12 (38%)	22 (6.3%)
	Proportion of persons who committed suicide attempt with a psychiatric diagnosis	30 (88.2%)	11 (91.6%)	17 (85%)	14 (87.5%)	16 (88.8%)	12 (100%)	18 (81.8%)
Suicide attempts – during imprisonment		10 (2.6%)	5 (5.5%)	5 (2.2%)	3 (2.0%)	7 (3.0%)	3 (9.4%)	7 (2.0%)
	Proportion of persons who committed suicide attempts before	5 (50%)	3 (60%)	2 (40%)	1 (33.3%)	4 (57.1%)	3 (100%)	2 (28.6%)
	Proportion of persons committed suicide attempt with psychiatric diagnosis	10 (100%)	5 (100%)	5 (100%)	3 (100%)	7 (100%)	3 (100%)	7 (100%)

Note. N = sample size (percentage). Percentages always refer to the total sample size of the respective group.

Table 5 - Total burden of mental and chronic somatic illnesses

		**Total disease burden (mental and somatic combined)**<br>(Number and percentage of people with at least one diagnosis)	**Total mental health burden**<br>(Number and percentage of people with at least one diagnosis)	**Total somatic chronic health burden**<br>(Number and percentage of people with at least one diagnosis)	**Dual Diagnosis burden**<br>(Number and percentage of people with at least one substance use diagnosis and another mental health comorbidity)
**Entire sample (N = 384)**		**253 (66%)**<br>***M*=2.28 (SD=2.44)**	**200 (52%)**<br>***M*=1.07 (SD=1.26)**	**164 (33%)**<br>***M*=1.22 (SD=1.89)**	**68 (18%)**
Gender (NA=3)	Female	28 (87%)<br>*M*=4.13 (SD=3.06)	26 (81%)<br>*M*=1.41 (SD=0.91)	22 (69%)<br>*M*=2.72 (SD=2.76)	3 (9.4%)
	Male	225 (64%)<br>*M*=2.11 (SD=2.44)	174 (50%)<br>*M*=1.03 (SD=1.28)	142 (41%)<br>*M*=1.08 (SD=1.73)	65 (19%)
Age (NA=0)	50 and older	120 (97%)<br>*M*=3.12 (SD=2.44)	97 (64%)<br>*M*=1.37 (SD=1.34)	86 (57%)<br>*M*=1.75 (SD=2.12)	30 (20%)
	Under 50	133 (57%)<br>*M*= 1.00 (SD=2.26)	103 (44%)<br>*M*=0.87 (SD=1.16)	78 (33%)<br>*M*=0.87 (SD=1.63)	38 (16%)
Sentence Type (NA=68)	Indefinite	84 (92.3%)<br>*M*=3.61 (SD=2.28)	77 (85%)<br>*M*=1.93 (SD=1.26)	46 (51%)<br>*M*=1.67 (SD=2.12)	25 (27%)
	Definite	138 (61%)<br>*M*=1.99 (SD=2.37)	108 (48%)<br>*M*=0.92 (SD=1.18)	92 (41%)<br>*M*=1.07 (SD=1.76)	68 (18%)

Note. N = sample size (percentage). Percentages always refer to the total sample size of the respective group. *M* = mean. SD = Standard Deviation
