# Supplementary figures and images for: Somatic and psychiatric health burden of male and female older incarcerated adults in Switzerland: a retrospective cross-sectional study
Source: BMJ Public Health. 2026 Jun 25;4(2):e004164. doi: 10.1136/bmjph-2025-004164 (PMC13358338; doi:10.1136/bmjph-2025-004164)

Figure 2 - Relationship between age and chronic somatic disease burden

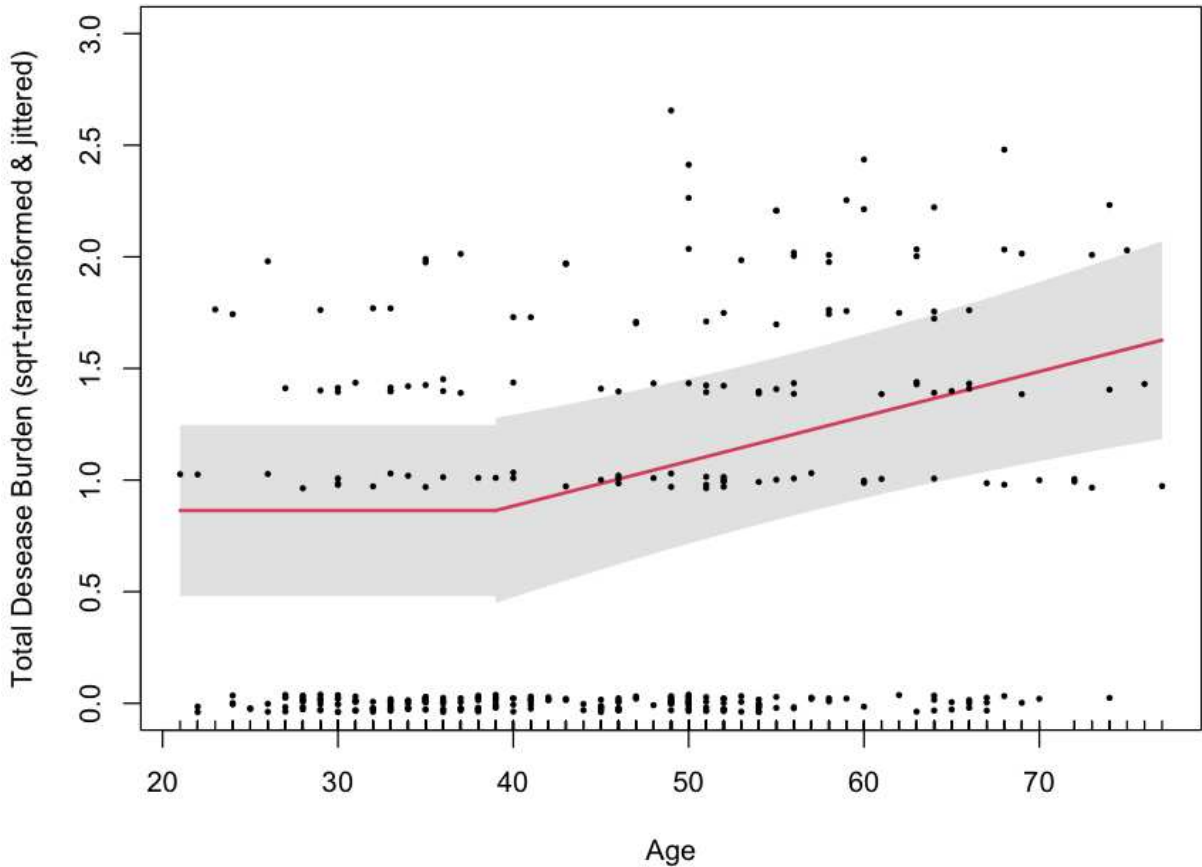

Supplement: Supplementary data [file bmjph-4-2-s003.pdf]
